# Tomography of the 2016 Kumamoto earthquake area and the Beppu-Shimabara graben

**DOI:** 10.1038/s41598-018-33805-0

**Published:** 2018-10-19

**Authors:** Dapeng Zhao, Kei Yamashita, Genti Toyokuni

**Affiliations:** Department of Geophysics, Tohoku University, Sendai, 980-8578 Japan

## Abstract

Detailed three-dimensional images of P and S wave velocity and Poisson’s ratio (σ) of the crust and upper mantle beneath Kyushu in SW Japan are determined, with a focus on the source area of the 2016 Kumamoto earthquake (M 7.3) that occurred in the Beppu-Shimabara graben (BSG) where four active volcanoes and many active faults exist. The 2016 Kumamoto earthquake took place in a high-velocity and low-σ zone in the upper crust, which is surrounded and underlain by low-velocity and high-σ anomalies in the upper mantle. This result suggests that, in and around the source zone of the 2016 Kumamoto earthquake, strong structural heterogeneities relating to active volcanoes and magmatic fluids exist, which may affect the seismogenesis. Along the BSG, low-velocity and high-σ anomalies do not exist everywhere in the upper mantle but mainly beneath the active volcanoes, suggesting that hot mantle upwelling is not the only cause of the graben. The BSG was most likely formed by joint effects of northward extension of the Okinawa Trough, westward extension of the Median Tectonic Line, and hot upwelling flow in the mantle wedge beneath the active volcanoes.

## Introduction

**Figure 1:**
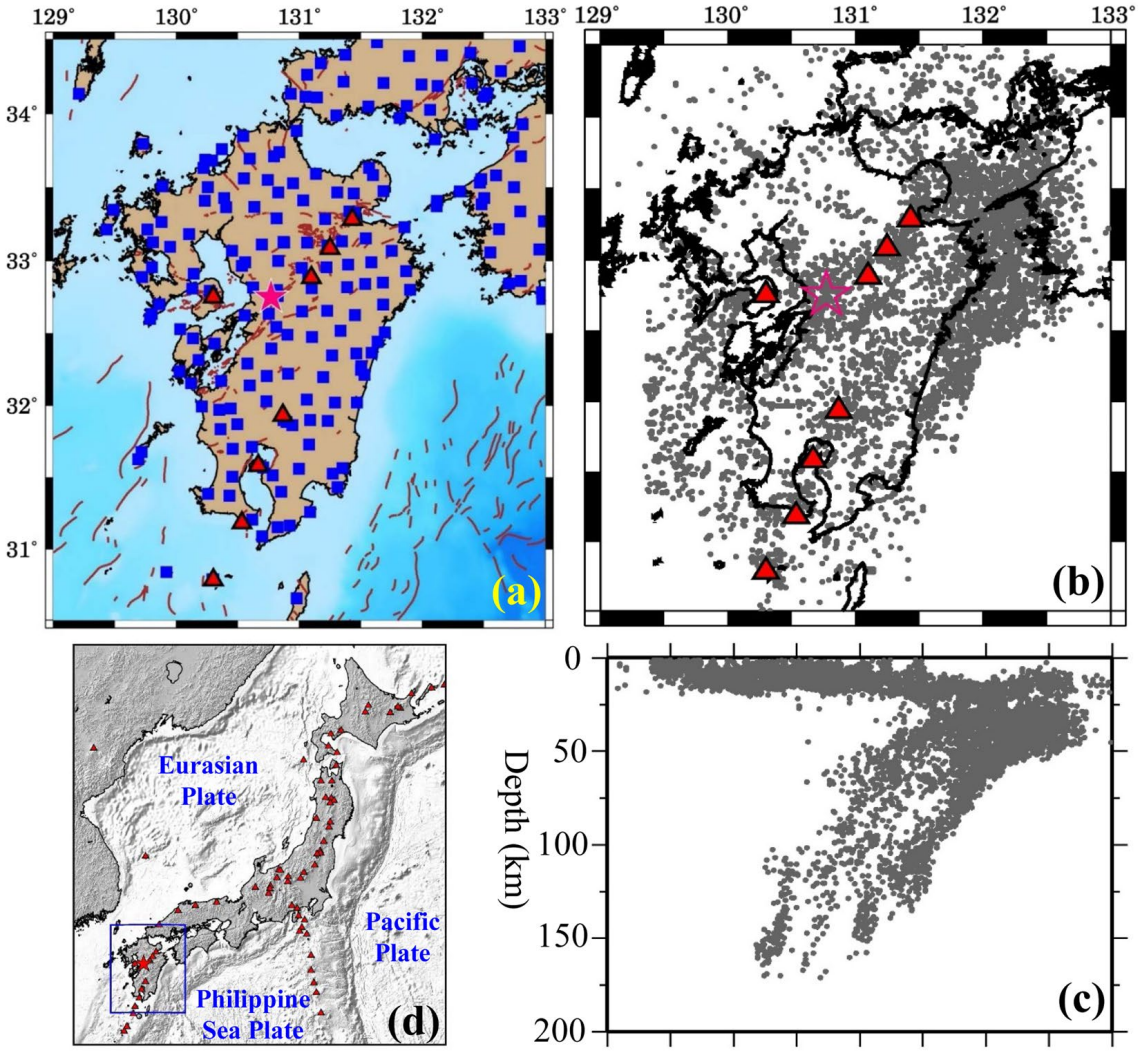
(**a**) Distribution of 206 seismograph stations (blue squares) used in this work. The brown lines denote active faults. (**b**) Map view and (**c**) east-west vertical cross-section showing the hypocentral distribution of 6002 local earthquakes (gray dots) used in this study. (**d**) Map of the Japan Islands and East Asia. The blue box shows the present study region enlarged in (**a,b**). The red triangles denote active volcanoes. The red star in (**a,b**) denotes the mainshock epicenter of the 2016 Kumamoto earthquake (M 7.3). This figure was generated using the Generic Mapping Tools^69^ version 4.5.8 (http://gmt.soest.hawaii.edu).

**Figure 2:**
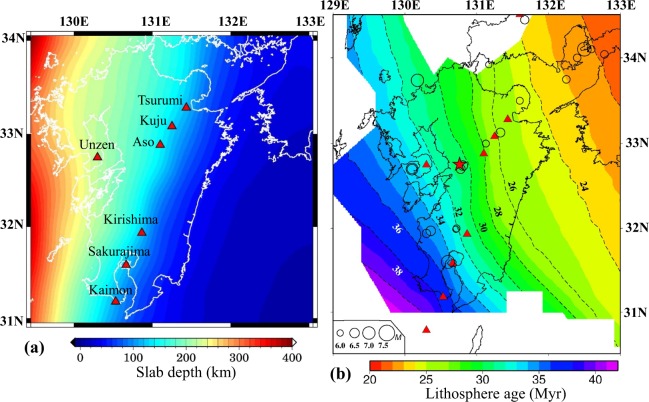
(**a**) Depth distribution of the upper interface of the subducting Philippine Sea slab in the study region. Its depth scale is shown at the bottom. The red triangles denote active arc volcanoes. The white lines denote the coastlines. (**b**) Distribution of the lithosphere age of the subducting Philippine Sea slab, whose scale is shown at the bottom. The open circles denote large crustal earthquakes (M ≥ 6.0; Depth < 30 km) during 1900 to 2017. The earthquake magnitude scale is shown at the lower-left corner. This figure was generated using the Generic Mapping Tools^69^ version 4.5.8 (http://gmt.soest.hawaii.edu).

Kyushu is one of the four major islands of Japan, in addition to Hokkaido, Honshu and Shikoku (Fig. 1). The young Philippine Sea (PHS) plate is subducting northwestward beneath the Eurasian plate along the Nankai Trough and the Ryukyu Trench at a rate of 4–5 cm/year, forming a mature subduction zone in Kyushu^1,2^. Figure 2a shows the geometry of the subducting PHS slab compiled from models of local earthquake tomography^3^ and teleseismic tomography^4^. The lithospheric age of the subducting PHS slab^5,6^ ranges from 24 Myr in NE Kyushu to 38 Myr in SW Kyushu (Fig. 2b). The lateral variations of the slab age may affect the arc magmatism and the generation of large crustal earthquakes^5^ and low-frequency microearthquakes^6^. Because of the active plate subduction, seismic and volcanic activities are very intense in Kyushu. During the past century, different types of earthquakes have taken place in the region, including megathrust earthquakes, such as the 1968 Hyuganada (M 7.5) earthquake, and inland crustal events, such as the 2005 western Fukuoka (M 7.0) earthquake^5,7^. Several active volcanoes exist in Kyushu, which form a clear volcanic front roughly along the 100-km depth contour of the subducting PHS slab and parallel with the Ryukyu Trench axis (Fig. 2a). A prominent active volcano, Unzen, is located in the back-arc area of Kyushu (Fig. 2), which erupted on 3 June 1991 and caused 44 fatalities^7^.

A unique tectonic feature of Kyushu is that the central part of the island is subject to north-south extensional stress, whereas the crust of the Japan Islands is generally affected by compressive regional stress^8,9^. Extensional deformation runs east-west across central Kyushu, resulting in the Beppu-Shimabara graben (BSG) where four active volcanoes (Tsurumi, Kuju, Aso and Unzen) exist, in particular, Mt. Aso with a large caldera whose area is ~400 km^2^. A number of M6 class historical earthquakes occurred in and around the BSG^7,10^ that is under a heterogeneous stress regime associated with both right-lateral faults and normal faults whose tensional axes trend north-south^8^. Concerning the driving force of the extensional deformation in central Kyushu, two hypotheses have been proposed: northward extension of the opening Okinawa Trough^11^, and transient variations in the convergence direction of the PHS plate^12^. However, the detailed structure and origin of the BSG are still not very clear.

The 2016 Kumamoto earthquake sequence wrote a new chapter of active seismotectonics and subduction dynamics in Kyushu (Fig. 1). This earthquake sequence, taking place in the upper crust of the BSG, began with a strong foreshock (M 6.5 in the Japan Meteorological Agency (JMA) scale) at local time 21:26 on 14 April 2016 and another big foreshock (M 6.4) at 00:03 on 15 April in the central part of Kumamoto Prefecture. Its mainshock (M 7.3; at 12 km depth) occurred at 01:25 on 16 April in the vicinity of the two foreshocks, which was followed by thousands of aftershocks (M 1.0–5.9) to date^7^. The Kumamoto earthquakes were caused by ruptures of the Futagawa and Hinagu fault zones which are representative faults in Kyushu and form the southern boundary of the BSG^13^. This earthquake sequence has extended toward the southwest and the northeast, reaching Oita Prefecture in NE Kyushu, indicating that most of the BSG has been activated that is composed of many active faults^13,14^. The closest volcano to the mainshock, Mt. Aso, has been more active before and after the Kumamoto earthquake, in particular, it erupted on 8 October 2016 (i.e., about 6 months after the Kumamoto mainshock), suggesting that the volcano and the earthquakes have been interacting with each other^15^. The 2016 Kumamoto earthquake caused 49 fatalities, over 1000 injured, and serious damage to the local infrastructures in Kumamoto and adjacent areas^7^.

Since the occurrence of the 2016 Kumamoto earthquake, many researchers have investigated the local seismicity and rupture process of the mainshock (e.g., Yagi *et al*.^16^, Yue *et al*.^17^ and the references therein), whereas only a few studies were made to investigate the three-dimensional (3-D) seismic structure of the source area to understand the relationship between the crustal structure and tectonic processes that generated the Kumamoto earthquake sequence. Wang *et al*.^18^ used P and S wave amplitude spectra of local earthquakes in the crust and the subducting PHS slab, including some Kumamoto aftershocks, to determine 3-D P and S wave attenuation (Qp and Qs) tomography down to 120 km depth beneath the Kumamoto source zone. Using a similar approach, Komatsu *et al*.^19^ obtained Qp and Qs tomographic images down to ~40 km depth beneath central-north Kyushu using local events that occurred during 2002 to 2012, i.e., their data set does not include the 2016 Kumamoto aftershocks. Shito *et al*.^20^ used arrival-time data of the Kumamoto aftershocks and other local crustal events during January 1996 to August 2016 to determine P and S wave velocity (Vp, Vs) tomography of the upper crust down to 20 km depth. Wang *et al*.^21^ used arrival-time data of the first P waves and the Moho-reflected PmP waves from local crustal earthquakes to determine 3-D Vp tomography of the crust in the Kumamoto source area. These studies have generally shown that the 2016 Kumamoto earthquake occurred in a high-velocity (high-V) and low-attenuation (high-Q) zone in the upper crust, whereas low-velocity (low-V) and high-attenuation (low-Q) anomalies exist in the lower crust and upper (or uppermost) mantle beneath the source area.

In this work, we utilized a large number of arrival-time data of local shallow and intermediate-depth earthquakes including many Kumamoto aftershocks to determine detailed 3-D Vp, Vs and Poisson’s ratio (*σ*) images of the crust and upper mantle down to 100 km depth beneath the entire Kyushu Island, with a focus on the 2016 Kumamoto source area and the BSG. Our results provide new insight into the structural heterogeneity in the crust and upper mantle beneath the BSG and its influence on the generation of the 2016 Kumamoto earthquake sequence, in particular, the effects of magmatic fluids and earthquake-volcano interactions in Kyushu. This study also sheds new light on the formation mechanism of the BSG.

## Data

We used arrival-time data of local earthquakes recorded by the dense seismic network deployed on the Japan Islands, which is composed of permanent stations of the JMA Seismic Network, the Japan University Seismic Network, and the High-Sensitivity Seismic Network^22^. Since October 1997 seismograms recorded by these seismic networks have been transmitted to and processed by JMA to monitor seismic activity in and around Japan^22^. Arrival times of P and S waves are measured from the three-component seismograms with an automatic processing system and monitored by the network staff daily. The arrival-time data are used to determine hypocentral parameters for each local earthquake. The resulting data base for the hypocentral parameters as well as the P and S wave arrival times is known as the JMA unified earthquake catalogue^22^.

In this study, we selected a best set of events (Fig. 1) from all the local earthquakes in and around Kyushu during June 2002 to November 2017 from the JMA unified earthquake catalogue. The seismicity is very active in Kyushu, but the event distribution is very inhomogeneous^7,8^. To select a best set of events with a homogeneous hypocentral distribution required for tomographic inversion, we divided the study volume into many small cubic blocks. Among all the earthquakes in each block, only the best event is selected that has the maximum number of P and S wave arrivals and the smallest error in the hypocentral parameters. After several tries we found that the cubic blocks with a size of 10 km × 10 km × 1 km lead to an optimal data set with enough events. As a result, 6002 earthquakes are selected that took place in the crust and the subducting PHS slab beneath the study region and were recorded at 206 seismic stations (Fig. 1). These events generated 179,951 P and 166,719 S wave arrival times that are used in the tomographic inversion. Note that many of the P and S wave arrivals in our data set were collected by staff of our Tohoku University, who picked P and S wave arrivals at all the stations that recorded each earthquake, in particular, at stations with large epicentral distances. In contrast, the JMA unified catalogue contains mainly P and S wave arrivals recorded at close stations for the purpose of routine earthquake location. The JMA arrival-time data have been double-checked by the research staff of our Tohoku University. The P-wave arrivals are measured from the vertical-component seismograms, whereas the S-wave arrivals are picked at horizontal-component seismograms. In our selected data set, the accuracy of the arrival times is estimated to be 0.05–0.15 s for P-waves and 0.10–0.15 s for S-waves. The arrivals with larger errors are not used in this study. We have tried to select the data set with different sizes of cubic blocks, and used different data sets to conduct tomographic inversions. The results show that the obtained 3-D velocity models are essentially the same^23^.

## Results

**Figure 3:**
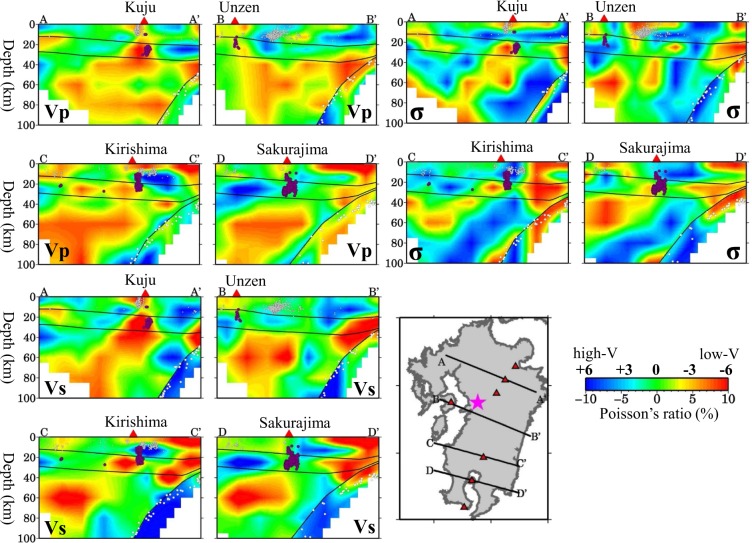
Vertical cross-sections of tomography along the four profiles shown in the inset map. The upper-left four panels show P-wave tomography (Vp), the lower-left four panels show S-wave tomography (Vs), and the upper-right four panels show Poisson’s ratio (*σ*). The red colors denote low velocity and high-σ, whereas the blue colors denote high velocity and low-σ, whose scales (in %) are shown beside the map. Areas with resolution <0.5 are masked in white. The Vp and Vs perturbations are relative to the 1-D velocity model shown in Fig. S4. The σ perturbations are relative to its average value (0.25). The red triangles denote active volcanoes. The red and white circles denote low-frequency micro-earthquakes and background seismicity, respectively, which occurred within a 10-km width of each profile during April 2002 to April 2016. The three black lines in each cross-section denote the Conrad and Moho discontinuities and the upper boundary of the subducting Philippine Sea slab. The red star in the map shows the mainshock epicenter of the 2016 Kumamoto earthquake (M 7.3). This figure was generated using the Generic Mapping Tools^69^ version 4.5.8 (http://gmt.soest.hawaii.edu).

**Figure 4:**
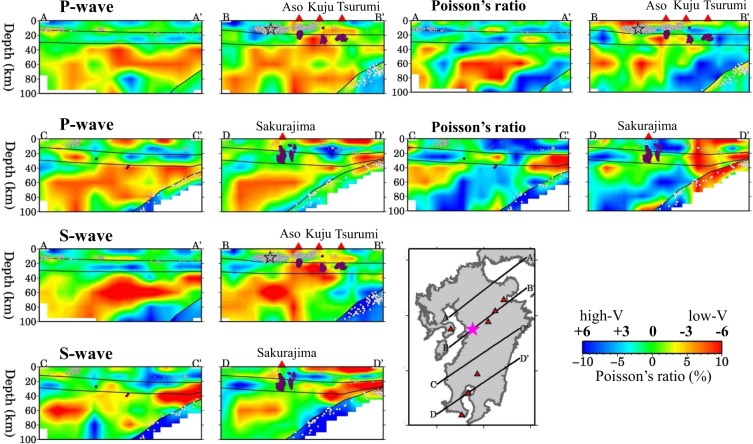
The same as Fig. 3 but along another four profiles shown in the inset map. The black open star in the cross-section B-B’ denotes the hypocenter of the 2016 Kumamoto earthquake (M 7.3). This figure was generated using the Generic Mapping Tools^69^ version 4.5.8 (http://gmt.soest.hawaii.edu).

**Figure 5:**
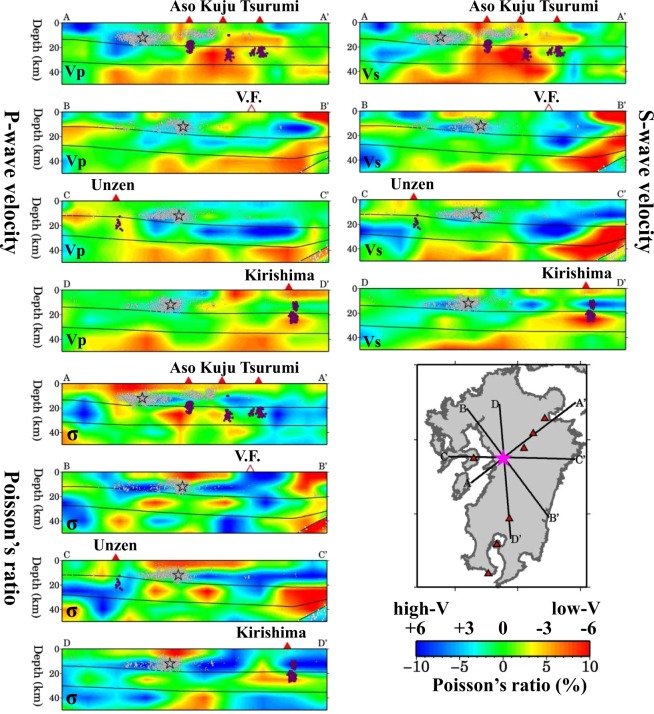
The same as Fig. 3 but along another four profiles shown in the inset map. The black open star denotes the hypocenter of the 2016 Kumamoto earthquake (M 7.3). The open red triangle atop the cross-section B-B’ denotes the location of the volcanic front (V.F.). This figure was generated using the Generic Mapping Tools^69^ version 4.5.8 (http://gmt.soest.hawaii.edu).

**Figure 6:**
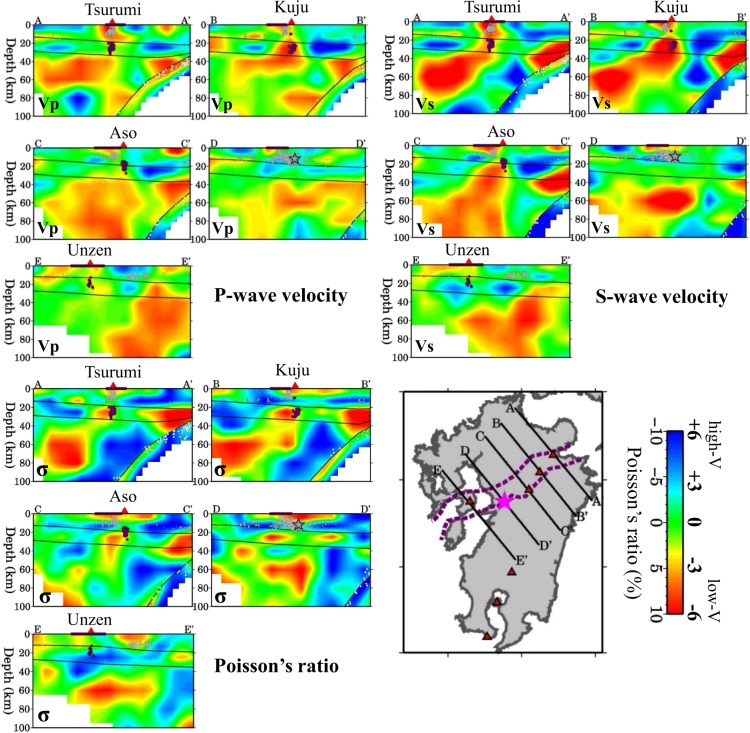
The same as Fig. 3 but along another five profiles shown in the inset map. The purple dotted lines in the map and the purple bar atop the cross-sections denote the range of the Beppu-Shimabara graben. The black open star in the cross-section D-D’ denotes the hypocenter of the 2016 Kumamoto earthquake (M 7.3). This figure was generated using the Generic Mapping Tools^69^ version 4.5.8 (http://gmt.soest.hawaii.edu).

Map views of the obtained 3-D Vp and Vs models and the resultant Poisson’s ratio (*σ*) images at depths of 4–100 km are shown in Supplementary Figs S1–S3. Vertical cross-sections of the Vp, Vs and *σ* images along different profiles are shown in Figs 3–6. In these figures, the Vp and Vs perturbations are relative to the 1-D velocity model shown in Fig. S4 with the Conrad and Moho geometries shown in Fig. S5. The subducting PHS slab is generally imaged as a significant high-V zone where intermediate-depth earthquakes occur frequently (Figs 3 and 4). However, in some cross-sections (Figs 3 and 4), the top part of the PHS slab exhibits a lower velocity and a higher *σ*, which may reflect the subducting oceanic crust containing abundant fluids^24,25^. Under the volcanic front and back-arc areas, prominent low-V and high-*σ* anomalies are revealed in the crust and mantle wedge (Figs 3 and 4), which may reflect hot and wet upwelling material caused by joint effects of fluids from the PHS slab dehydration and mantle-wedge convection driven by the plate subduction^2,18,25–27^. The hot and wet upwelling material forms the source of arc magma feeding the arc and back-arc volcanoes in Kyushu^18,28^. In the forearc mantle wedge, low-V and high-*σ* anomalies are also visible (Figs 3 and 4), but they do not reflect hot magmas, because the temperature is not high in the forearc and there is no active volcano there (Fig. 2), instead they reflect the forearc mantle serpentinization due to abundant fluids from the young and warm PHS slab^2,24,25^.

Figure 5 shows four vertical cross-sections of Vp, Vs and*σ* images passing through the hypocenter of the 2016 Kumamoto mainshock. We can see that the mainshock occurred in a high-V and low-*σ* zone in the upper crust, whereas the lower crust right beneath the hypocenter exhibits average or slightly lower velocities but a high-*σ*. In the uppermost mantle under the mainshock hypocenter, Vp and Vs are very low, and*σ* is higher (Fig. 5). In the areas adjacent to the mainshock hypocenter, prominent low-V and high-*σ* anomalies exist in the crust and upper mantle, in particular, under the active Aso and Unzen volcanoes (Fig. 5). Figure 6 shows five vertical cross-sections of Vp, Vs and*σ* images across the BSG. A low-V and high-*σ* anomaly exists in the upper mantle beneath the graben, and the anomaly extends upward to the crust under the active volcanoes (Fig. 6).

Detailed resolution analyses are made to examine the reliability and spatial resolution of the tomographic images. The results show that the above-mentioned main features are robust and reliable (for details, see Supplementary Figs S6–S15).

## Discussion

Many geophysical and geochemical studies^1,2,24,28,29^ have investigated the arc magmatism and volcanism in Kyushu. Close to the source zone of the 2016 Kumamoto earthquake, there exist Unzen and Aso volcanoes which are among the most active volcanoes in Japan. The latest eruption sequence of Unzen^7^ began on 17 November 1990. The Aso volcano erupted recently on 8 October 2016, which is considered to be related to the 2016 Kumamoto earthquake^15^. Prominent low-V and high-*σ* zones are revealed under the volcanic front and back-arc area (Figs 3 and 4). The low-V and high-*σ* anomalies are visible continuously or intermittently in the crust and mantle wedge, which may reflect hot and wet upwelling material driven by the PHS plate subduction^2,28^. Low-frequency micro-earthquakes (M 0.0–2.2), which are associated with upward migration of fluids and arc magma from the mantle wedge to the crust^30,31^, took place in or around the low-V and high-*σ* zones in the lower crust and uppermost mantle beneath the active volcanoes (Fig. 5). The low-V and high-*σ* anomalies revealed by our velocity tomography correspond very well to the low-Q zones revealed by seismic attenuation tomography^18,19^ and high-conductivity anomalies detected by electromagnetic imaging^32^.

The existence of low-V, low-Q and high-*σ* anomalies in the crust and mantle wedge under active volcanoes is a general seismological feature of subduction zones (see a recent review by Zhao^33^). Hydrous minerals in the subducting slab break down with the increasing pressure and temperature, and fluids released to the mantle wedge trigger arc magmatism by hydrous melting^27,34^. Geochemical studies have shown that volcanic rocks erupted from the Aso volcano have a greater contamination with slab-derived fluids than the other volcanoes in the BSG^1,29^, which is consistent with our result showing that the low-V and high-*σ* anomaly under the Aso volcano is the most significant (Fig. 4).

Low-V and high-*σ* anomalies also appear in the forearc mantle wedge and lower crust (Figs 3 and 4), which also exhibit strong attenuation (low-Q) in the Q tomography^18^. The low-V, low-Q and high-σ zones reflect the forearc mantle serpentinization due to the presence of abundant fluids from the dehydration of the young and warm PHS slab (Fig. 2), as pointed out by several previous studies of seismic velocity^2,24,35^ and attenuation^18,19,36^ in Kyushu. Serpentinization is a common feature in the forearc mantle wedge when abundant fluids are released from a young and warm slab^37–39^. In general, a large volume of fluid exists in the forearc mantle wedge where the temperature is not high, which is the condition of serpentinization^25,40^. Christensen^40^ showed a relation between serpentinization and seismic velocity as well as Poisson’s ratio. A low-V, low-Q and high-σ anomaly has been revealed in the forearc mantle wedge of many subduction zones^33^, such as central Japan^37^, Cascadia^38,41^, and beneath the serpentinite seamounts in the Mariana forearc^42^. However, some researchers found that the forearc mantle in some regions exhibits high-V and/or high-Q^36,43^ or moderate V or Q^44^, where in general an old plate is subducting. Obviously, the relationship between seismic velocity/attenuation and serpentinization in different subduction zones cannot be attributed to the same causes. Wang *et al*.^18^ revealed a higher Qp/Qs in the Kyushu forearc, which provides another piece of evidence for serpentinization, because a large volume of fluid in the serpentinized mantle wedge may increase Qp/Qs^45^.

Earthquake generation is governed by the state of stress and strength on a fault^8,16,17^. In the past two decades, accumulating pieces of evidence from many studies with various approaches have shown that fluids play an important role in the nucleation of large earthquakes^5,46–48^. Experimental studies have confirmed that fluids can significantly affect the frictional strength and the stability of sliding of a fault^49,50^. Under the maximum horizontal compressive stress with the nearly east-west direction^8,51,52^, many normal faults have been developed in and around the BSG (Fig. 1a), providing a fundamental condition for the earthquake generation. Our result show that the 2016 Kumamoto earthquake took place in a high-V and low-σ zone in the upper crust, whereas low-V and high-σ anomalies exist in the uppermost mantle beneath the Kumamoto source area (Fig. 5). A low-resistivity zone is revealed north of the Futagawa fault, extending to at least 20 km depth and apparently bounded by the fault^53^. This low-resistivity anomaly may reflect the existence of fluids. Values of ^3^He/^4^He are higher in the fault area^8,53^, which also implies a path for transporting fluids from the upper mantle to the surface. Fluids can reduce the friction coefficient and so the strength of a fault, and so can induce earthquakes^8,46^. The nucleation-patch size of the Kumamoto earthquake may be on the order of 1–2 km or even smaller^54–57^, whereas the lateral resolution of our tomography is ~20 km and its vertical resolution is 9–12 km in the source zone (Fig. S8). However, our results indicate that, in and around the source zone of the 2016 Kumamoto earthquake, strong structural heterogeneities relating to active volcanoes and magmatic fluids exist, which may affect the seismogenesis in the Kumamoto area. From this point of view, the causal mechanism of the Kumamoto earthquake seems similar to that of many large crustal earthquakes in Japan, in particular, those taking place nearby an active volcano, such as the 2000 western Tottori earthquake (M 7.3) and the 2016 central Tottori earthquake (M 6.6) which occurred near the Daisen volcano in western Honshu^5^, as well as the 2008 Iwate-Miyagi earthquake (M 7.2) which occurred near the Kurikoma volcano in NE Japan^58^.

The tectonics of SW Japan has undergone complex and significant changes in the past 15 Myr, being dominated by interactions between the PHS and Eurasian plates, whereas the major driver of the tectonics has been the ever-evolving subduction history of the PHS plate^59–62^. The PHS plate is composed of two segments with significantly different ages separated by the Kyushu Palau Ridge: the Eocene-Cretaceous West Philippine Basin segment subducting at the Ryukyu Trench and beneath South Kyushu, and the younger (27–15 Myr) Shikoku Basin segment subducting at the Nankai Trough. The age difference of the two segments has affected the slab dip angle of each segment, which in turn has caused the complexity of the tectonic and volcanic evolution of SW Japan. By 15 Ma, back-arc rifting in the Japan Sea and spreading in the Shikoku Basin had ceased. The PHS plate began to subduct northwards beneath the Eurasian plate at ~15 Ma. Some researchers proposed that the northward subduction of the PHS plate ceased during 10–6 Ma^59^, whereas others suggested that there was no late Miocene halt in the PHS plate subduction^60^ or the subduction stagnated at another period (14–8 Ma)^62^. In Kyushu, features of the current subduction regime began around 6 Ma, and the subduction direction changed from the north to NNW^61^, which was coincident with the start of back-arc rifting in the Okinawa Trough. Southern Kyushu underwent ~30 degrees of anticlockwise rotation after ~6 Ma, which facilitated rifting in the BSG region^59^. North of the Median Tectonic Line (MTL), a rectangular volcano-tectonic depression of ~70 km long and ~40 km wide began forming at ~6 Ma^59^. From ~2 Ma to present, Kyushu has entered its latest tectonic phase, and the PHS plate has shifted its subduction direction from NNW to NW, which resulted in an increased dextral component of subduction, leading to intensified dextral motion along the MTL^59^. Current tectonics in Kyushu is dominated by ~72–79 mm/year convergence of the PHS and Eurasian plates at a slightly oblique (right-lateral sense) angle^59^. Strike-slip and extensional faulting is ongoing in the BSG. Toward the southwest, along-strike of the BSG, active back-arc rifting continues to occur in the Okinawa Trough^59–62^.

Along the BSG, although low-V and high-σ anomalies are generally visible in the lower crust and uppermost mantle, the anomalies do not exist everywhere in the upper mantle but mainly beneath the active volcanoes (Fig. 6 and Supplementary Figs S1–S3). Under the non-volcanic areas of the graben, the low-V and high-σ anomalies are not significant in the crust and upper mantle (Fig. 6). These results suggest that hot mantle upwelling seems not the main cause of this rift zone. The southern boundary of the BSG, the so-called Oita-Kumamoto tectonic line, is considered to be westward extension of the MTL^63^. The MTL, together with the Northern Chugoku shear zone^64^ or the Southern Japan Sea fault zone^65^ along the Japan Sea coast, are mainly caused by the oblique subduction of the PHS plate beneath the Eurasian plate, resulting in the right-lateral strike-slip motions along these tectonic lines and shear zones^64,65^. From this viewpoint, the BSG is considered to be a passive rift rather than an active rift that is produced mainly by hot mantle upwelling or plumes^66^. However, because the BSG is located in the volcanic arc and back-arc areas where active volcanoes and magmatic fluids exist and the temperature is generally higher, the Eurasian plate becomes much thinner and mechanically much weaker in the BSG than that in Shikoku and central Japan. Thus, it is easier for the MTL to extend to northern Kyushu, being driven by the oblique subduction of the PHS plate. Taking into account all the previous results and the present findings, we deem that the BSG is produced by the joint effect of three factors: (1) northward extension of the opening Okinawa Trough; (2) westward extension of the MTL; and (3) hot and wet upwelling flow in the mantle wedge beneath the active volcanoes (Tsurumi, Kuju, Aso and Unzen) in North-Central Kyushu.

## Conclusion

High-resolution tomographic images of P and S wave velocity and Poisson’s ratio of the crust and upper mantle under Kyushu are determined using a large number of high-quality arrival-time data of local earthquakes. Our results provide new insight into the seismotectonics in Kyushu and the formation of the BSG. New findings of this study are summarized as follows.

1. The 2016 Kumamoto mainshock occurred in a high-V and low-σ zone in the upper crust underlain by low-V and high-*σ* anomalies in the upper mantle, indicating that in and around the Kumamoto source zone, strong structural heterogeneities relating to active volcanoes and magmatic fluids exist, which may have affected the seismogenesis in the BSG.
2. Along the BSG, low-V and high-σ anomalies do not exist everywhere in the upper mantle but mainly beneath the active volcanoes, suggesting that hot mantle upwelling is not the main cause of this rift zone.
3. The formation of the BSG was caused by joint effects of the northward extension of the opening Okinawa Trough, westward extension of the MTL, and hot and wet upwelling in the mantle wedge beneath active volcanoes.

## Methods

We applied a tomographic method^4,26^ to our travel-time data to determine 3-D Vp and Vs models beneath Kyushu. A 3-D grid is arranged in the study volume. Hypocentral parameters of the local earthquakes and velocity perturbations at the grid nodes from an initial velocity model (Supplementary Fig. S4) are taken to be unknown parameters. The perturbation of seismic velocity at any point in the study volume is computed by linearly interpolating the velocity perturbations at the eight grid nodes surrounding that point. Following the previous works of the study region^2,18,28,36^, depth variations of the Conrad and Moho boundaries (Supplementary Fig. S5) and the upper interface of the subducting PHS slab (Fig. 2a) are introduced into the model, since the three velocity boundaries beneath western Japan are found to exist and their geometries have been well determined by previous studies^25,33,67^. When these curved velocity discontinuities are included in the starting model, travel times and ray paths can be computed more precisely^26,33^. A 3-D ray tracing technique^26^ is applied to calculate theoretical travel times and ray paths. Elevations of seismic stations and the surface topography are considered in the 3-D ray tracing. The LSQR algorithm^68^ with damping regularization is applied to solve the system of observation equations that relate the arrival-time data to the hypocentral and velocity parameters^33^. The local events are relocated in the inversion process, and their hypocentral uncertainties do not exceed 2 km, since all the events took place under or very close to the dense seismic network (Fig. 1). After the 3-D Vp and Vs models are determined, a model of Poisson’s ratio (*σ*) is obtained^33,46^ with the formula

$$\,{({\rm{Vp}}/{\rm{Vs}})}^{2}=2(1-{\rm{\sigma }})/(1-2{\rm{\sigma }}).$$

Our optimal 3-D Vp and Vs models (Supplementary Figs S1 and S2) have a lateral grid interval of 0.2° and a vertical grid interval of 9–20 km. The P-wave root-mean-square (RMS) travel-time residuals before and after the inversion are 0.252 s and 0.196 s, respectively, and the corresponding S-wave RMS residuals are 0.334 s and 0.294 s, respectively. The variance reductions of the P and S wave data are 54% and 44%, respectively.

## Electronic supplementary material

Supplementary Information

## Data Availability

The datasets generated and/or analysed during the current study are available from the corresponding author on reasonable request.

## References

[1] Kita I (2001). Contemporaneous ascent of within-plate type and island-arc type magmas in the Beppu–Shimabara graben system, Kyushu island, Japan. J. Volcanol. Geotherm. Res..

[2] Zhao D, Asamori K, Iwamori H (2000). Seismic structure and magmatism of the young Kyushu subduction zone. Geophys. Res. Lett..

[3] Nakajima J, Hirose F, Hasegawa A (2009). Seismotectonics beneath the Tokyo metropolitan area, Japan: Effect of slab-slab contact and overlap on seismicity. J. Geophys. Res..

[4] Zhao D (2012). Imaging the subducting slabs and mantle upwelling under the Japan Islands. Geophys. J. Int..

[5] Zhao D, Liu X, Hua Y (2018). Tottori earthquakes and Daisen volcano: Effects of fluids, slab melting and hot mantle upwelling. Earth Planet. Sci. Lett..

[6] Hua Y, Zhao D, Xu Y, Liu X (2018). Age of the subducting Philippine Sea slab and mechanism of low-frequency earthquakes. Geophys. Res. Lett..

[7] Japan Meteorological Agency (JMA): http://www.jma.go.jp.

[8] Matsumoto S (2018). Prestate of stress and fault behavior during the 2016 Kumamoto earthquake (*M* 7.3). Geophys. Res. Lett..

[9] Kato A, Nakamura K, Hiyama Y (2016). The 2016 Kumamoto earthquake sequence. Proc. Japan Acad. Ser. B.

[10] Usami, T. *Materials for Comprehensive List of Destructive Earthquakes in Japan*. Univ. Tokyo Press, Tokyo (2003).

[11] Nakahigashi K (2004). Seismic structure of the crust and uppermost mantle in the incipient stage of back arc rifting—northernmost Okinawa Trough. Geophys. Res. Lett..

[12] Itoh Y, Kusumoto S, Takemura K (2014). Evolutionary process of Beppu Bay in central Kyushu, Japan: a quantitative study of the basinforming process controlled by plate convergence modes. Earth Planets Space.

[13] Toda S (2016). Slip-partitioned surface ruptures for the Mw 7.0 16 April 2016 Kumamoto, Japan earthquake. Earth Planets Space.

[14] Mochizuki K, Mitsui Y (2016). Crustal deformation model of the Beppu−Shimabara graben area, central Kyushu, Japan, based on inversion of three-component GNSS data in 2000–2010. Earth Planets Space.

[15] Ozawa T, Fujita E, Ueda H (2016). Crustal deformation associated with the 2016 Kumamoto Earthquake and its effect on the magma system of Aso volcano. Earth Planets Space.

[16] Yagi Y (2016). Rupture process of the 2016 Kumamoto earthquake in relation to the thermal structure around Aso volcano. Earth Planets Space.

[17] Yue H (2017). The2016 Kumamoto Mw = 7.0 earthquake: A significant event in a fault–volcano system. J. Geophys. Res..

[18] Wang Z, Zhao D, Liu X (2017). Seismic attenuation tomography of the source zone of the 2016 Kumamoto earthquake (M 7.3). J. Geophys. Res..

[19] Komatsu M, Takenaka H, Oda H (2017). Three-dimensional P- and S-wave attenuation structures around the source region of the 2016 Kumamoto earthquakes. Earth, Planets Space.

[20] Shito A (2017). Seismic velocity structure in the source region of the 2016 Kumamoto earthquake sequence, Japan. Geophys. Res. Lett..

[21] Wang H (2018). Crustal tomography of the 2016 Kumamoto earthquake area in West Japan using P and PmP data. Geophys. J. Int..

[22] Okada Y (2004). Recent progress of seismic observation networks in Japan – Hi-net, F-net, K-NET and KiK-net. Earth Planets Space.

[23] Yamashita, K. *Anisotropic tomography of the 2016 Kumamoto earthquake area and the lowermost mantle*. Master’s Thesis, Tohoku University, 194 pp. (2018).

[24] Xia S, Zhao D, Qiu X (2008). Tomographic evidence for the subducting oceanic crust and forearc mantle serpentinization under Kyushu, Japan. Tectonophysics.

[25] Abe Y, Ohkura T, Hirahara K, Shibutani T (2013). Along-arc variation in water distribution in the uppermost mantle beneath Kyushu, Japan, as derived from receiver function analyses. J. Geophys. Res..

[26] Zhao D, Hasegawa A, Horiuchi S (1992). Tomographic imaging of P and S wave velocity structure beneath northeastern Japan. J. Geophys. Res..

[27] Iwamori H, Zhao D (2000). Melting and seismic structure beneath the northeast Japan arc. Geophys. Res. Lett..

[28] Wang Z, Zhao D (2006). Vp and Vs tomography of Kyushu, Japan: New insight into arc magmatism and forearc seismotectonics. Phys. Earth Planet. Inter..

[29] Miyoshi M, Fukuoka T, Sano T, Hasenaka T (2008). Subduction influence of Philippine Sea plate on the mantle beneath northern Kyushu, SW Japan: An examination of boron contents in basaltic rocks. J. Volcanol. Geotherm. Res..

[30] Hasegawa, A. & Zhao, D. Deep structure of island arc magmatic regions as inferred from seismic observations. *Magmatic Systems* (edited by M. P. Ryan), Academic Press, pp. 179–195 (1994).

[31] Aso N, Ohta K, Ide S (2013). Tectonic, volcanic, and semi-volcanic deep low-frequency earthquakes in western Japan. Tectonophysics.

[32] Hata M (2017). 3-D electrical resistivity structure based on geomagnetic transfer functions exploring the features of arc magmatism beneath Kyushu, Southwest Japan Arc. J. Geophys. Res..

[33] Zhao, D. *Multiscale Seismic Tomograph*y. Springer, 304 pp., New York (2015).

[34] Horiuchi S, Iwamori H (2016). A consistent model for fluid distribution, viscosity distribution, and flow-thermal structure in subduction zone. J. Geophys. Res..

[35] Tahara M (2008). Seismic velocity structure around the Hyuganada region, Southwest Japan, derived from seismic tomography using land and OBS data and its implications for interplate coupling and vertical crustal uplift. Phys. Earth Planet. Inter..

[36] Liu X, Zhao D (2015). Seismic attenuation tomography of the Southwest Japan arc: New insight into subduction dynamics. Geophys. J. Int..

[37] Kamiya S, Kobayashi Y (2000). Seismological evidence for the existence of serpentinized wedge mantle. Geophys. Res. Lett..

[38] Hyndman R, Peacock S (2003). Serpentinization of the forearc mantle. Earth Planet. Sci. Lett..

[39] Alt J, Shanks W (2006). Stable isotope compositions of serpentinite seamounts in the Mariana forearc: Serpentinization processes, fluid sources and sulfur metasomatism. Earth Planet. Sci. Lett..

[40] Christensen NI (2004). Serpentinites, peridotites, and seismology. Int. Geol. Rev..

[41] Chen C, Zhao D, Wu S (2015). Tomographic imaging of the Cascadia subduction zone: Constraints on the Juan de Fuca slab. Tectonophysics.

[42] Pozgay S (2009). Seismic attenuation tomography of the Mariana subduction system: Implications for thermal structure, volatile distribution, and slow spreading dynamics. Geochem. Geophys. Geosyst..

[43] Bohm M, Haberland C, Asch G (2013). Imaging fluid-related subduction processes beneath central Java (Indonesia) using seismic attenuation tomography. Tectonophysics.

[44] Rychert C (2008). Strong along-arc variations in attenuation in the mantle wedge beneath Costa Rica and Nicaragua. Geochem. Geophys. Geosyst..

[45] Toksöz M, Johnston D, Timur A (1979). Attenuation of seismic waves in dry and saturated rocks: I. Laboratory measurements. Geophysics.

[46] Zhao D, Kanamori H, Negishi H, Wiens D (1996). Tomography of the source area of the 1995 Kobe earthquake: Evidence for fluids at the hypocenter?. Science.

[47] Tong P, Zhao D, Yang D (2012). Tomography of the 2011 Iwaki earthquake (M 7.0) and Fukushima nuclear power plant area. Solid Earth.

[48] Toyokuni G, Zhao D, Chen K (2016). Tomography of the source zone of the 2016 South Taiwan earthquake. Geophys. J. Int..

[49] Blanpied M (1998). Quantitative measure of the variation in fault rheology due to fluid-rock interactions. J. Geophys. Res..

[50] Tenthorey E, Cox S, Todd H (2003). Evolution of strength recovery and permeability during fluid–rock reaction in experimental fault zones. Earth Planet. Sci. Lett..

[51] Savage M (2016). Stress, strain rate and anisotropy in Kyushu, Japan. Earth Planet. Sci. Lett..

[52] Yoshida K (2016). Stress rotations due to the M6.5 foreshock and M7.3 mainshock in the 2016 Kumamoto, SW Japan, earthquake sequence: Stress field after the M 7.3 earthquake. Geophys. Res. Lett..

[53] Aizawa K (2017). Seismicity controlled by resistivity structure: The 2016 Kumamoto earthquakes, Kyushu Island, Japan. Earth Planets Space.

[54] Andrews DJ (1976). Rupture propagation with finite stress in antiplane strain. J. Geophys. Res..

[55] Day SM (1982). Three-dimensional simulation of spontaneous rupture: the effect of nonuniform prestress. Bull. Seismol. Soc. Am..

[56] Uenishi K, Rice JR (2003). Universal nucleation length for slip weakening rupture instability under nonuniform fault loading. J. Geophys. Res..

[57] Galis M (2015). On the initiation of sustained slip-weakening ruptures by localized stresses. Geophys. J. Int..

[58] Zhao D, Kitagawa H, Toyokuni G (2015). A water wall in the Tohoku forearc causing large crustal earthquakes. Geophys. J. Int..

[59] Kamata H, Kodama K (1999). Volcanic history and tectonics of the Southwest Japan arc. Island Arc.

[60] Kimura J, Stern R, Yoshida T (2005). Reinitiation of subduction and magmatic responses in SW Japan during Neogene time. Geol. Soc. Am. Bull..

[61] Seno T (1989). Philippine Sea plate kinematics. Modern Geol..

[62] Taira A (2001). Tectonic evolution of the Japanese island arc system. Annu. Rev. Earth Planet. Sci..

[63] Kusumoto S (2016). Dip distribution of Oita–Kumamoto Tectonic Line located in central Kyushu, Japan, estimated by eigenvectors of gravity gradient tensor. Earth Planets Space.

[64] Gutscher M, Lallemand S (1999). Birth of a major strike-slip fault in SW Japan. Terra Nova.

[65] Itoh Y, Tsutsumi H, Yamamoto H, Arato H (2002). Active right-lateral strike-slip fault zone along the southern margin of the Japan Sea. Tectonophysics.

[66] Logatchev N, Zorin Y, Rogozhina V (1983). Baikal rift: active or passive? Comparison of the Baikal and Kenya rift zones. Tectonophysics.

[67] Katsumata A (2010). Depth of the Moho discontinuity beneath the Japanese islands estimated by traveltime analysis. J. Geophys. Res..

[68] Paige C, Saunders M (1982). LSQR: An Algorithm for Sparse Linear Equations and Sparse Least Squares. ACM Trans. Math. Softw..

[69] Wessel P, Smith W (1998). New, improved version of Generic Mapping tools released. Eos Trans. AGU.

