# Supplementary material for: Tomography of the 2016 Kumamoto earthquake area and the Beppu-Shimabara graben
Source: Sci Rep. 2018 Oct 19;8:15488. doi: 10.1038/s41598-018-33805-0 (PMC6195613; doi:10.1038/s41598-018-33805-0)
Supplement: Supplementary file 1 — Supplementary Information [file 41598_2018_33805_MOESM1_ESM.pdf]

Tomography of the 2016 Kumamoto earthquake area and the Beppu-Shimabara graben

Supplementary Information

Dapeng Zhao, Kei Yamashita, Genti Toyokuni

Department of Geophysics, Tohoku University, Sendai 980-8578, Japan

Correspondence to: Dapeng Zhao (zhao@tohoku.ac.jp)

Abstract

This supplementary information contains the following materials: map views of the obtained P and S wave tomography (Figures S1 and S2), map views of the Poisson's ratio image (Figure S3), the average one-dimensional velocity model in the study region (Figure S4), depth distributions of the Conrad and Moho discontinuities in the study region (Figure S5), distribution of diagonal elements of the resolution matrix for P and S wave tomography (Figure S6), distribution of the standard errors of P and S wave velocity perturbations (Figure S7), results of checkerboard resolution tests (Figures S8 and S9), and results of restoring resolution tests in map views (Figures S10 and S11) and vertical cross-sections (Figures S12-S15).

Tomographic results

Figures S1 and S2 show map views of the optimal 3-D P and S wave velocity (Vp, Vs) models obtained by this study. Figure S3 shows map views of the Poisson's ratio image derived from the obtained Vp and Vs models (see the Methods for details). Active volcanoes and the epicenter of the 2016 Kumamoto earthquake (M 7.3) are also shown in the three figures.

One-dimensional velocity model

Figure S4 shows the average one-dimensional (1-D) velocity model in the study

region compiled from the previous studies (Zhao et al., 2000; Wang and Zhao, 2006; Xia et al., 2008; Liu and Zhao, 2015). This 1-D velocity model is taken as the starting model for the three-dimensional (3-D) tomographic inversions.

Seismic discontinuities

Many previous studies have shown that the Conrad and Moho discontinuities and the upper boundary of the subducting Philippine Sea (PHS) slab exist in and around the Kyushu region, and they are sharp seismic discontinuities from which reflected and converted waves are observed (e.g., Zhao et al. 2000; Katsumata, 2010; Abe et al., 2013; Zhao, 2015). Figure S5 shows geometries of the Conrad and Moho discontinuities derived from the model of Katsumata (2010). Figure 2a shows the geometry of the subducting PHS slab boundary derived from the results of local earthquake tomography (Nakajima et al., 2009) and teleseismic tomography (Zhao et al., 2012).

Resolution analysis

Detailed resolution analyses are made using our data set (Figure 1) and the tomographic method (Zhao et al., 1992; Zhao, 2015). The resolution matrix and covariance matrix are calculated using the methods described by Thurber (1983) and Zhao et al. (1992). Figure S6 shows distributions of the diagonal elements of the resolution matrix for P and S wave tomography. Figure S7 shows distributions of the standard errors of P and S wave velocity perturbations.

A direct and convenient way to evaluate the reliability of a tomographic model is to first calculate a set of synthetic travel-time data by tracing the rays of P and S wave data in a synthetic model, and then to compare the inversion result with the input synthetic model. In this study we performed extensive checkerboard resolution tests (Zhao et al., 1992; Zhao, 2015) to assess the adequacy of the ray coverage and to evaluate the tomographic resolution of the entire study region. To make a checkerboard, positive and negative velocity perturbations are assigned alternatively to the 3-D grid nodes that are arranged in the modeling space, the image of which is straightforward and easy to remember. Therefore, by looking at the result of the

synthetic inversion for the checkerboard, one can easily understand where the resolution is good and where it is poor. Before conducting the tomographic inversion, random noise (-0.2 s to +0.2 s) with a standard deviation of 0.1 s is added to the synthetic travel-time data to simulate the picking errors of the data.

Figures S8 and S9 show the results of two checkerboard resolution tests for P and S wave tomography in the study region. In one test (Fig. S8), the lateral grid interval is 0.2°, and the test results show that the checkerboard pattern is generally reconstructed at depths of 0-100 km, but the amplitude of velocity anomalies is not fully recovered at depths of 60-100 km. In another test (Fig. S9), the lateral grid interval is 0.5°, and the test results show that both the checkerboard pattern and amplitude of velocity anomalies are well reconstructed in the crust and upper mantle down to 100 km depth. These test results indicate that, in the study region, both the Vp and Vs models have a resolution of 0.2° in the crust and uppermost mantle (0-40 km), and the resolution is better than 0.5° in the upper mantle.

We also conducted restoring resolution tests (Zhao et al., 1992; Zhao, 2015) to confirm the main features of the obtained tomographic results. The procedure of the restoring tests is the same as that of the checkerboard tests, but the input model is derived from the obtained tomographic model (Figures S1 and S2 and Figs 3-6). Random noise (-0.2 s to +0.2 s) with a standard deviation of 0.1 s is added to the theoretical travel times calculated for the synthetic model. The test results (Figures S10-S15) show that main features of the tomographic images, such as the high-velocity subducting PHS slab and low-velocity anomalies in the crust and mantle wedge, are well recovered, indicating that those features are reliable and robust.

References

Abe, Y., Ohkura, T., Hirahara, K. & Shibutani, T. Along-arc variation in water distribution in the uppermost mantle beneath Kyushu, Japan, as derived from receiver function analyses. *J. Geophys. Res.* **118**, 3540–3556 (2013).

Katsumata, A. Depth of the Moho discontinuity beneath the Japanese islands estimated by traveltime analysis. *J. Geophys. Res*. **115**, B04303 (2010).

Liu, X. & Zhao, D. Seismic attenuation tomography of the Southwest Japan arc: New insight into subduction dynamics. *Geophys. J. Int.* **201**, 135-156 (2015).

Nakajima, J., Hirose, F. & Hasegawa, A. Seismotectonics beneath the Tokyo metropolitan area, Japan: Effect of slab-slab contact and overlap on seismicity. *J. Geophys. Res*. **114**, B08309 (2009).

Thurber, C. Earthquake locations and three-dimensional crustal structure in the Coyote Lake area, central California. *J. Geophys. Res*. **88,** 8226–8236 (1983).

Wang, Z. & Zhao, D. Vp and Vs tomography of Kyushu, Japan: New insight into arc magmatism and forearc seismotectonics. *Phys. Earth Planet. Inter*. **157**, 269-285 (2006).

Xia, S., Zhao, D., Qiu, X. Tomographic evidence for the subducting oceanic crust and forearc mantle serpentinization under Kyushu, Japan. *Tectonophysics* **449**, 85-96 (2008).

Zhao, D. *Multiscale Seismic Tomography*. Springer, 304 pp., New York (2015).

Zhao, D., Hasegawa, A. & Horiuchi, S. Tomographic imaging of P and S wave velocity structure beneath northeastern Japan. *J. Geophys. Res*. **97**, 19909–19928 (1992).

Zhao, D., Asamori, K. & Iwamori, H. Seismic structure and magmatism of the young Kyushu subduction zone. *Geophys. Res. Lett*. **27**, 2057–2060 (2000).

Zhao, D., Yanada, T., Hasegawa, A., Umino, N. & Wei, W. Imaging the subducting slabs and mantle upwelling under the Japan Islands. *Geophys. J. Int*. **190**, 816-828 (2012).

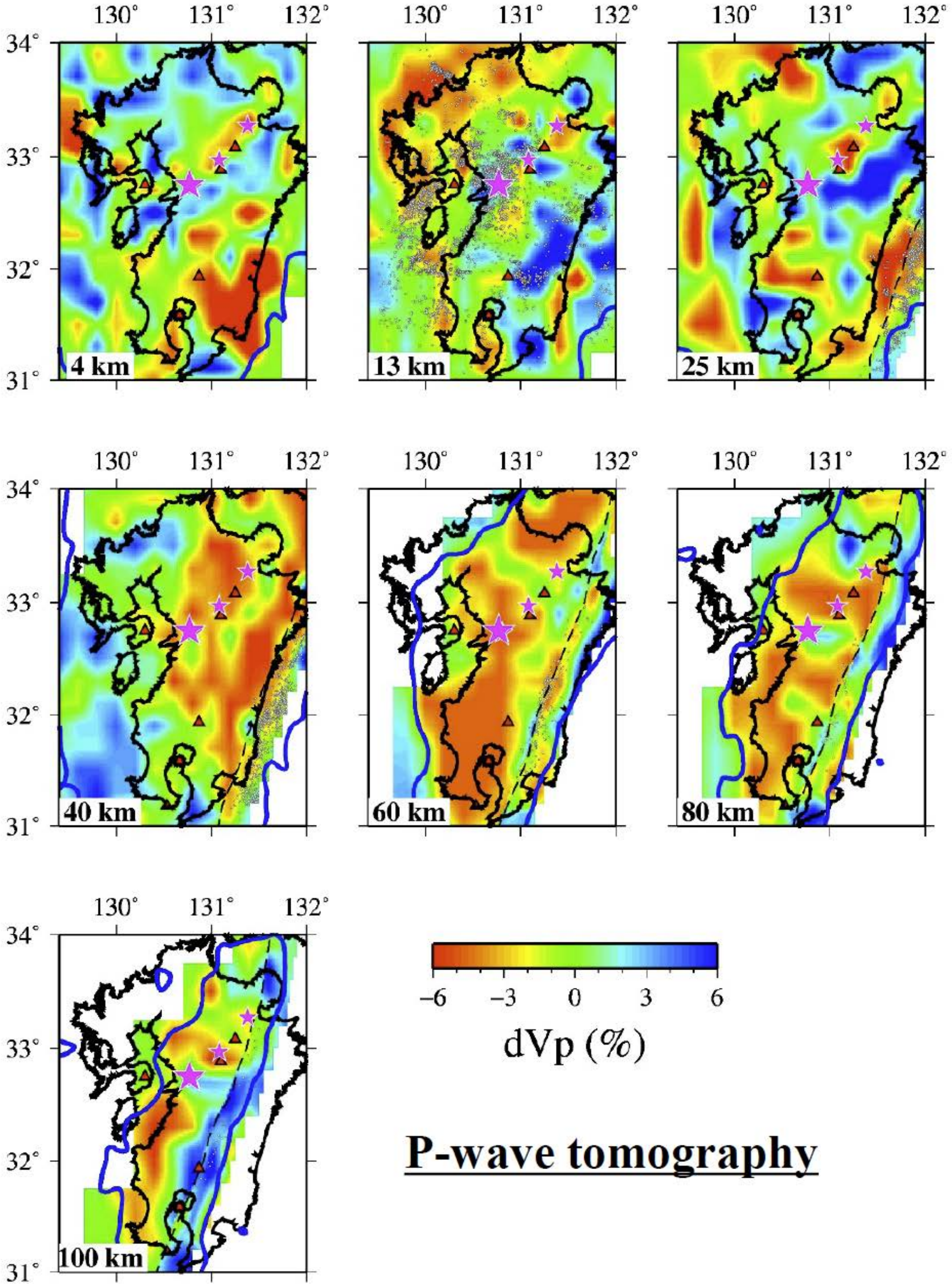

Fig. S1. Map views of P-wave tomography obtained by this study. The layer depth is shown at the lower-left corner of each map. The red and blue colors denote low and high velocity perturbations, respectively, whose scale (in %) is shown at the bottom. The velocity perturbations are relative to the 1-D velocity model shown in Fig. S4 with the Conrad and Moho geometries shown in Fig. S5. Areas with hit counts < 50 are masked in white. The blue contour lines mark the areas with resolution > 0.5. The red triangles denote active volcanoes. The purple stars denote epicenters of the 2016 Kumamoto mainshock (M 7.3) and its major aftershocks. The black dashed line denotes the location of the upper boundary of the subducting Philippine Sea slab at each depth. This figure was generated using the Generic Mapping Tools version 4.5.8 (http://gmt.soest.hawaii.edu).

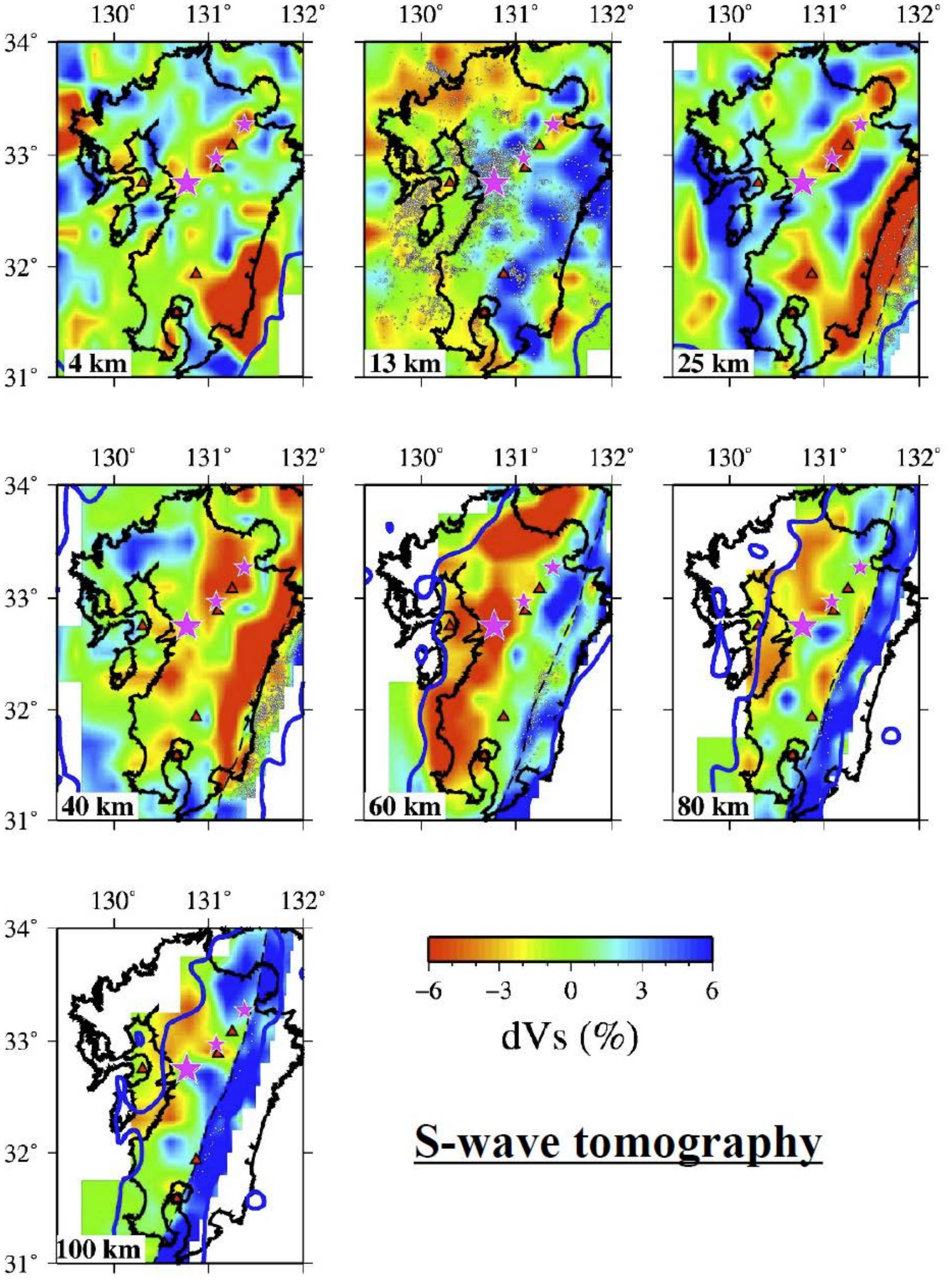

Fig. S2. The same as Fig. S1 but for S-wave tomography obtained by this study. This figure was generated using the Generic Mapping Tools version 4.5.8 (http://gmt.soest.hawaii.edu).

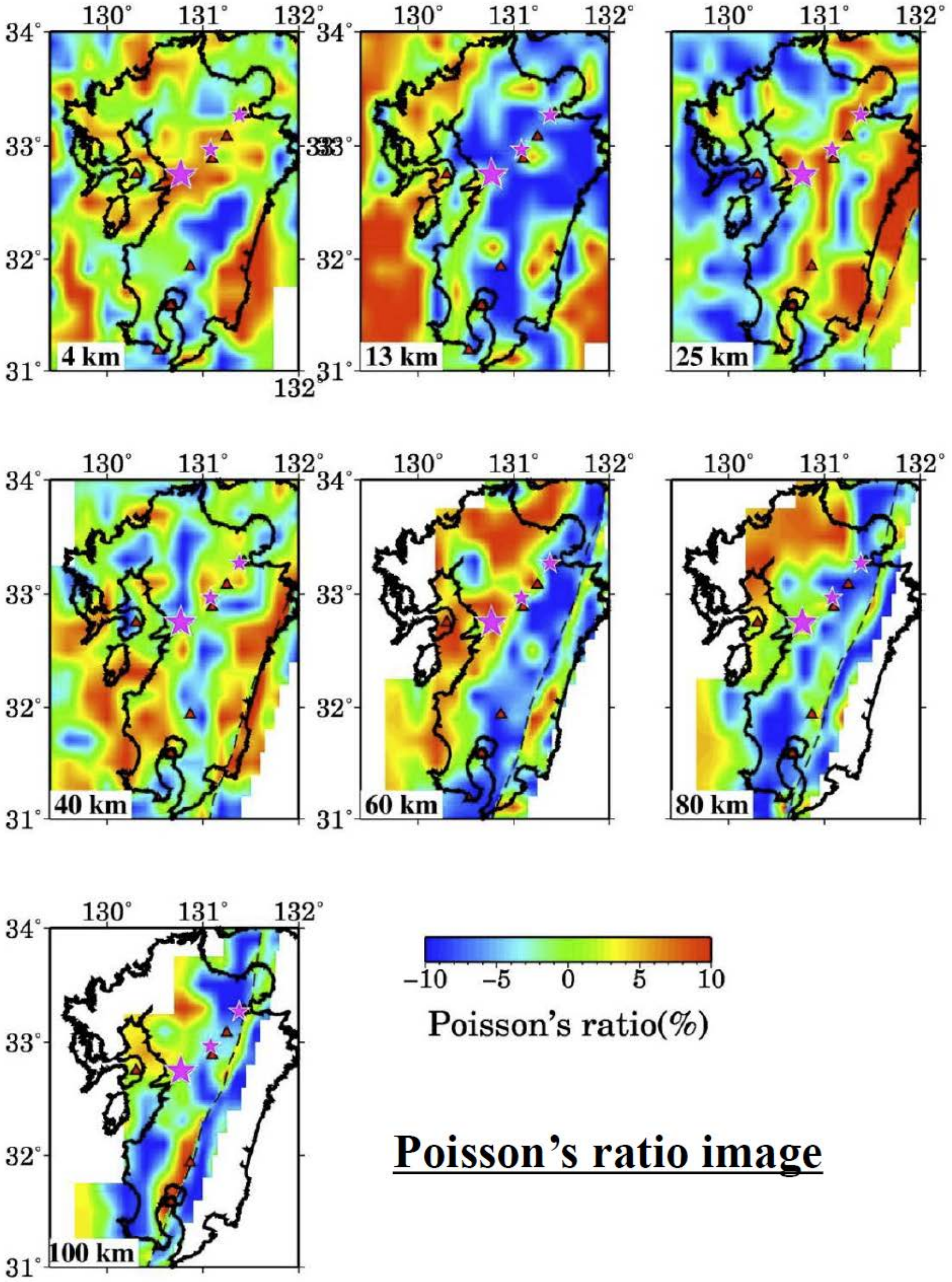

Fig. S3. The same as Fig. S1 but the Poisson's ratio images. This figure was generated using the Generic Mapping Tools version 4.5.8 (http://gmt.soest.hawaii.edu).

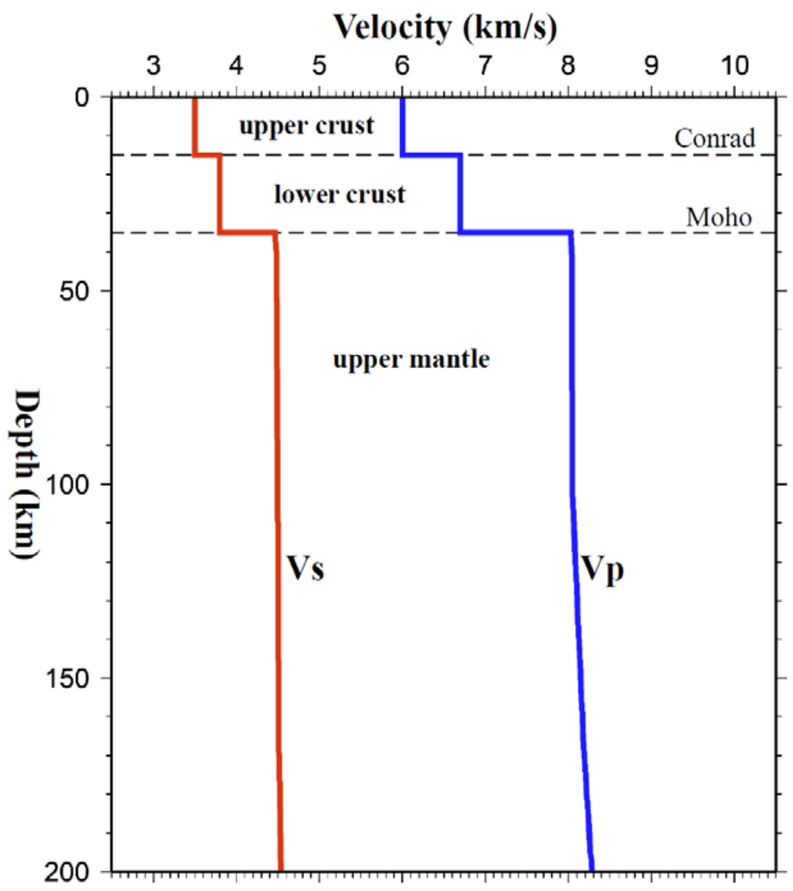

Fig. S4. The average one-dimensional (1-D) velocity model of the study region. The two dashed lines denote the Conrad and Moho discontinuities. This figure was generated using the Generic Mapping Tools version 4.5.8 (http://gmt.soest.hawaii.edu).

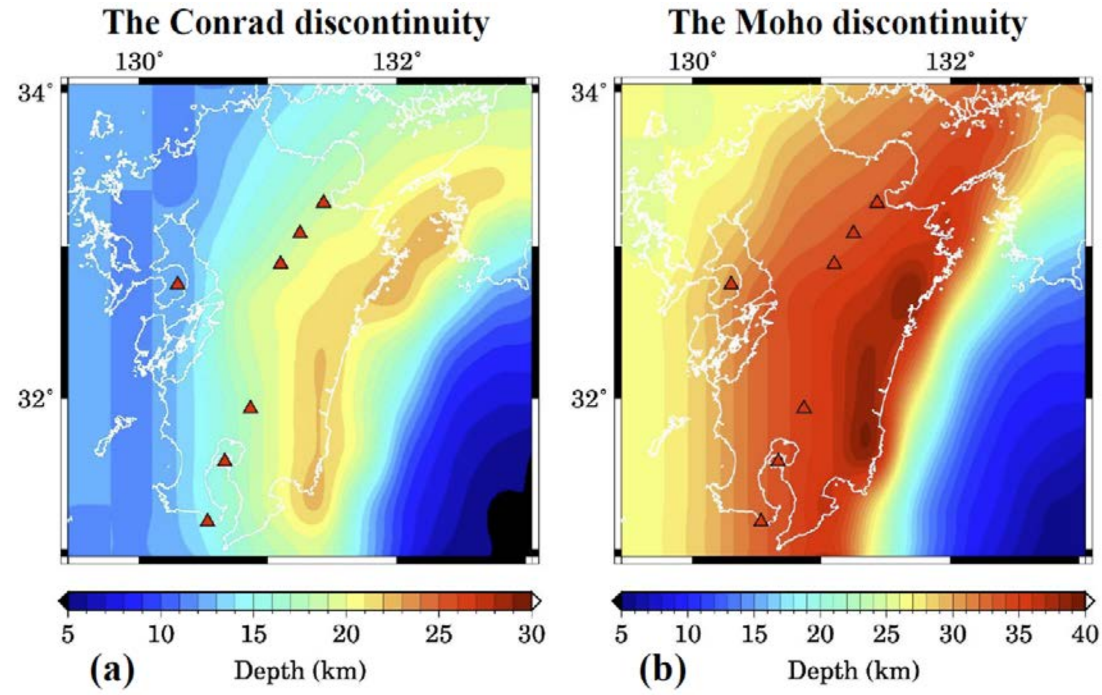

Fig. S5. Depth distribution of (a) the Conrad and (b) the Moho discontinuities in the study region. The depth scales are shown at the bottom. The red triangles denote active arc volcanoes. This figure was generated using the Generic Mapping Tools version 4.5.8 (http://gmt.soest.hawaii.edu).

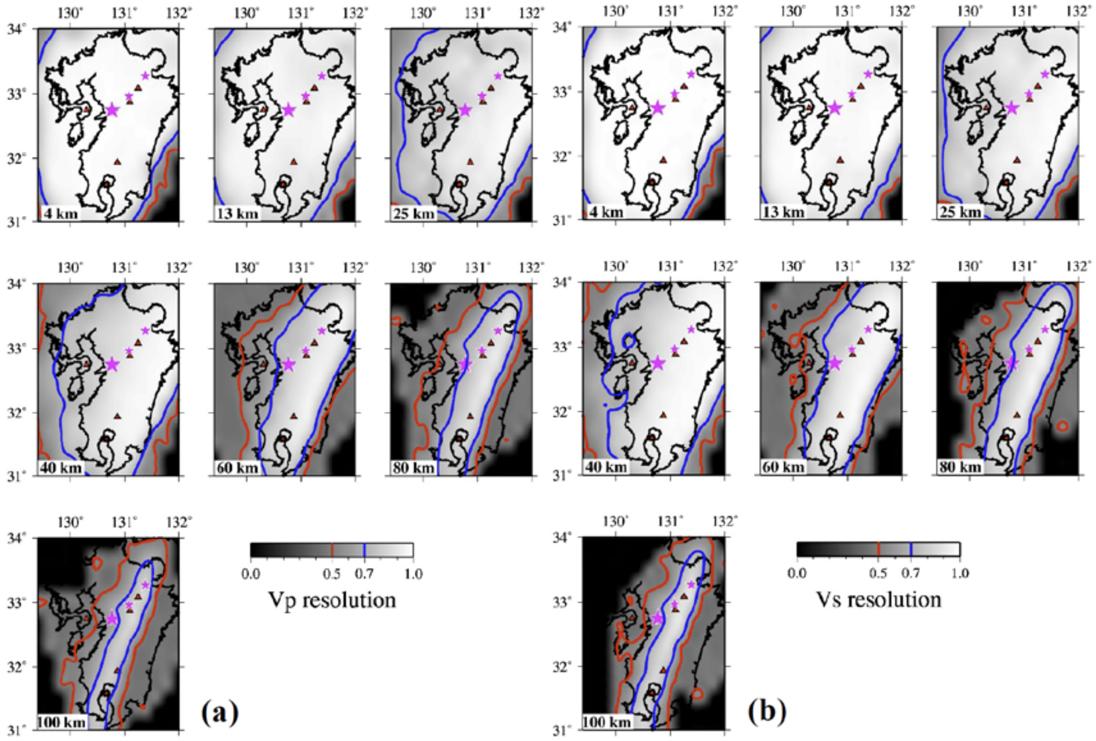

Fig. S6. Distribution of the diagonal elements of the resolution matrix for (a) P and (b) S wave velocity (Vp, Vs) tomography. The scales for the Vp and Vs resolutions are shown at the bottom. The layer depth is shown at the lower-left corner of each map. The red and blue contour lines mark the areas with the resolution greater than 0.5 and 0.7, respectively. The red triangles denote active volcanoes. The purple stars denote epicenters of the 2016 Kumamoto mainshock (M 7.3) and its major aftershocks. This figure was generated using the Generic Mapping Tools version 4.5.8 (http://gmt.soest.hawaii.edu).

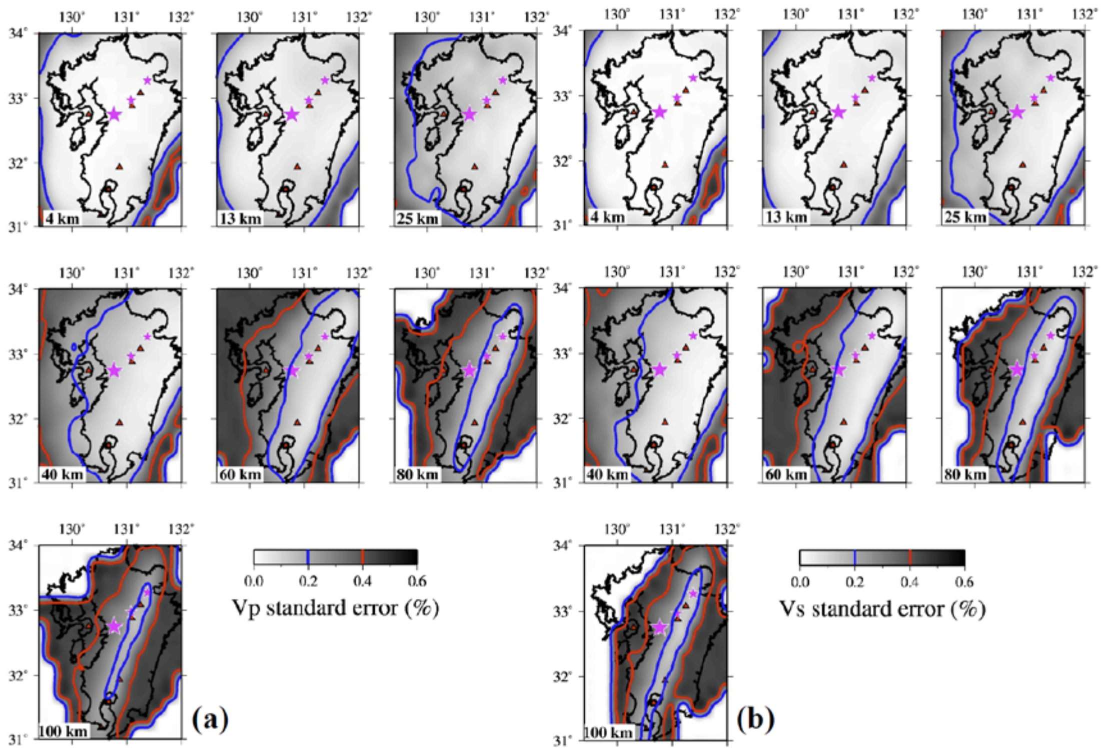

Fig. S7. Distribution of the standard errors (in %) of (a) P and (b) S wave velocity (Vp, Vs) perturbations. The scales for the Vp and Vs errors are shown at the bottom. The layer depth is shown at the lower-left corner of each map. The red and blue contour lines mark the areas with the errors smaller than 0.4% and 0.2%, respectively. The red triangles denote active volcanoes. The purple stars denote epicenters of the 2016 Kumamoto mainshock (M 7.3) and its major aftershocks. This figure was generated using the Generic Mapping Tools version 4.5.8 (http://gmt.soest.hawaii.edu).

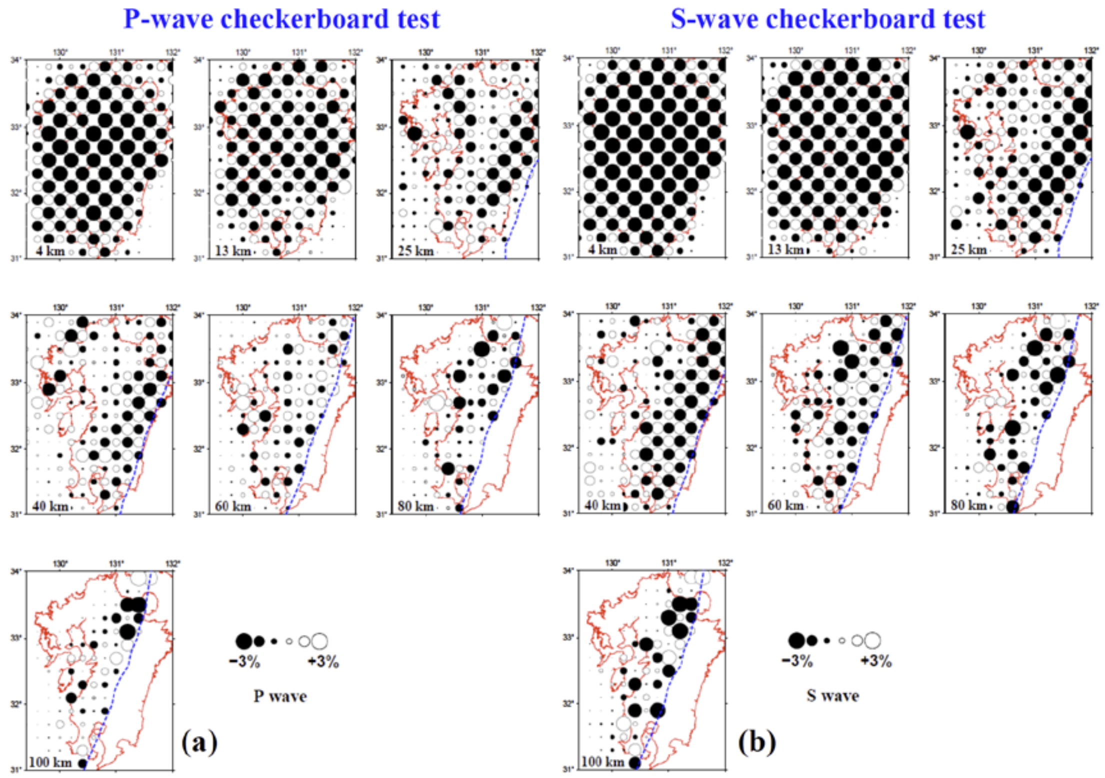

Fig. S8. Results of a checkerboard resolution test for (a) P and (b) S wave tomography. The lateral grid interval is 0.2° (~20 km). The layer depth is shown at the lower-left corner of each map. The solid and open circles denote low and high velocity perturbations (in %), respectively, whose scale is shown at the bottom. The red lines show the coastlines. The blue dashed line denotes the location of the upper boundary of the subducting Philippine Sea slab at each depth. This figure was generated using the Generic Mapping Tools version 4.5.8 (http://gmt.soest.hawaii.edu).

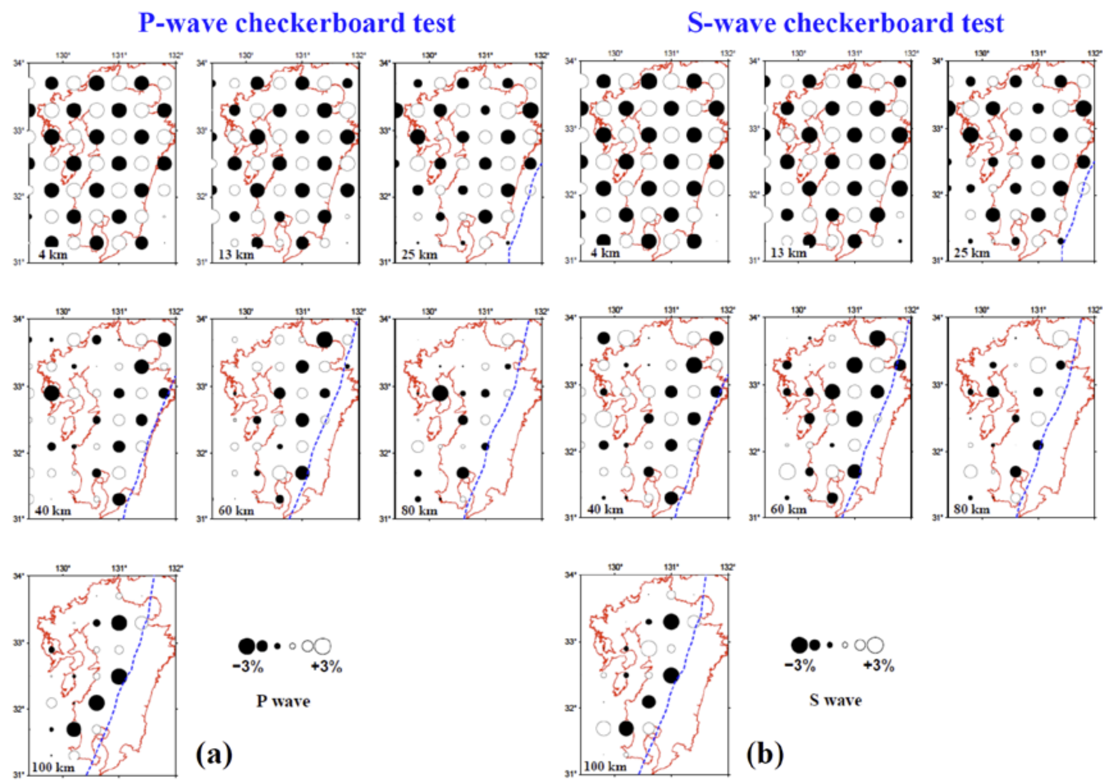

Fig. S9. The same as Fig. S8 but the lateral grid interval is 0.5° (~50 km). This figure was generated using the Generic Mapping Tools version 4.5.8 (http://gmt.soest.hawaii.edu).

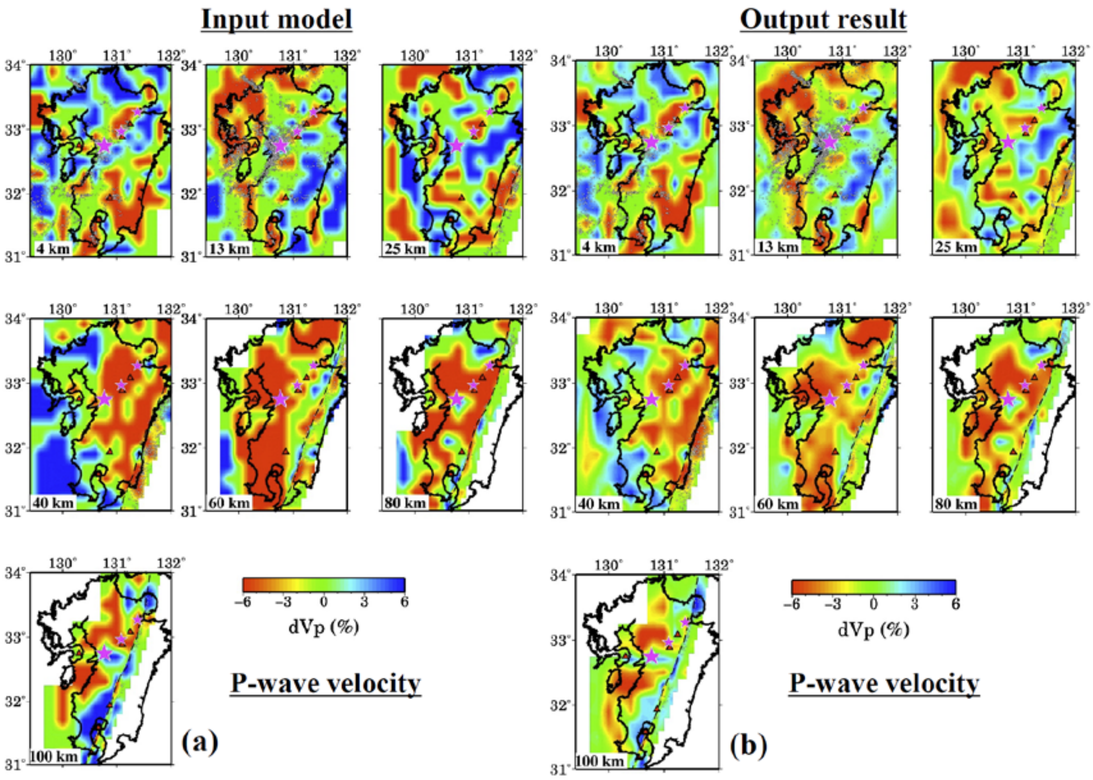

Fig. S10. The input model (a) and output result (b) of a restoring resolution test for P-wave tomography. The labels are the same as those in Fig. S1. This figure was generated using the Generic Mapping Tools version 4.5.8 (http://gmt.soest.hawaii.edu).

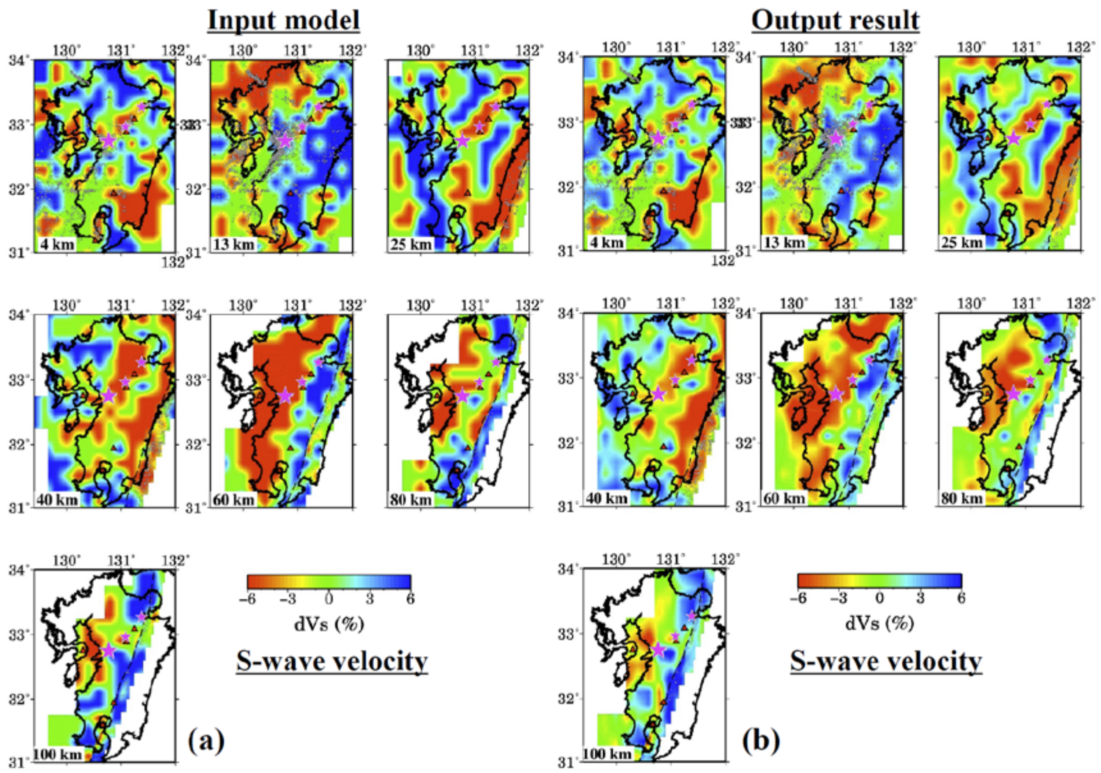

Fig. S11. The input model (a) and output result (b) of a restoring resolution test for S-wave tomography. The labels are the same as those in Fig. S2. This figure was generated using the Generic Mapping Tools version 4.5.8 (http://gmt.soest.hawaii.edu).

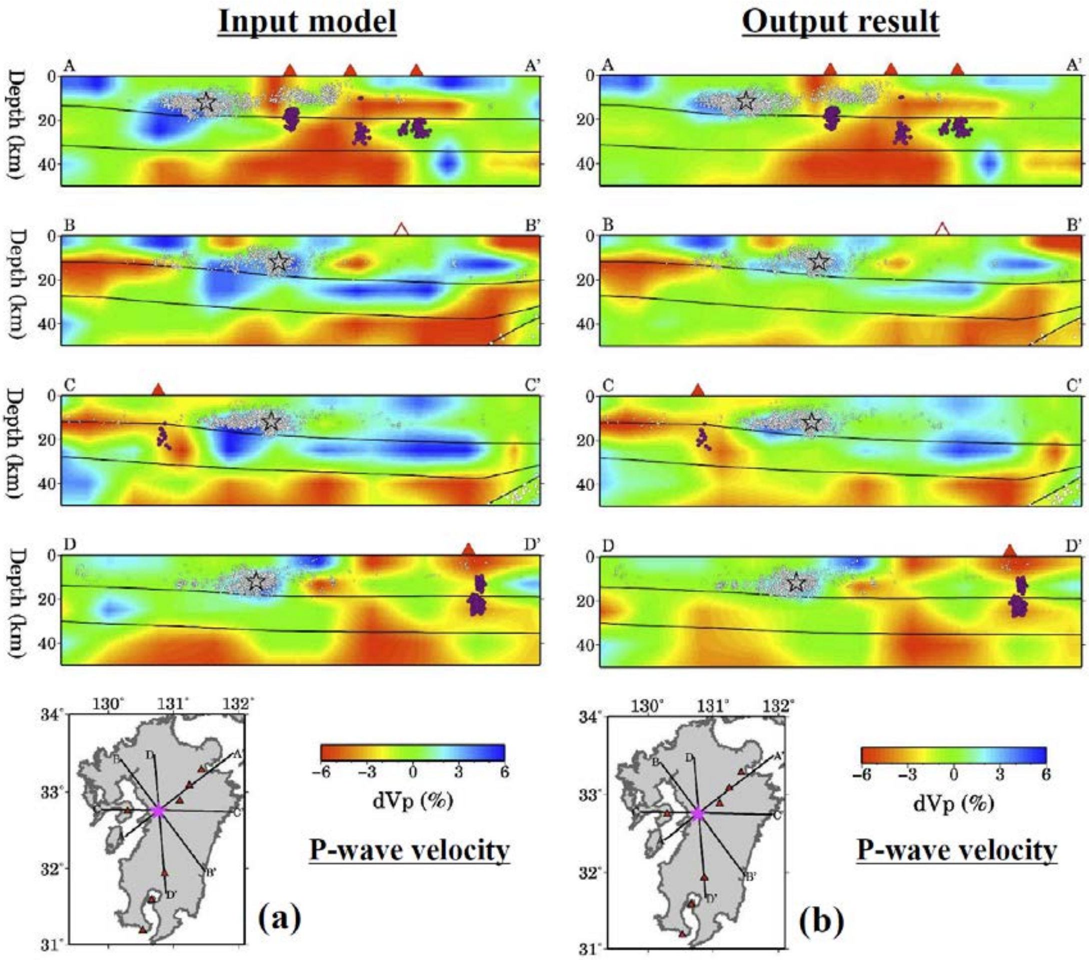

Fig. S12. The input model (a) and output result (b) of a restoring resolution test for P-wave tomography. The labels are the same as those in Fig. 5. This figure was generated using the Generic Mapping Tools version 4.5.8 (http://gmt.soest.hawaii.edu).

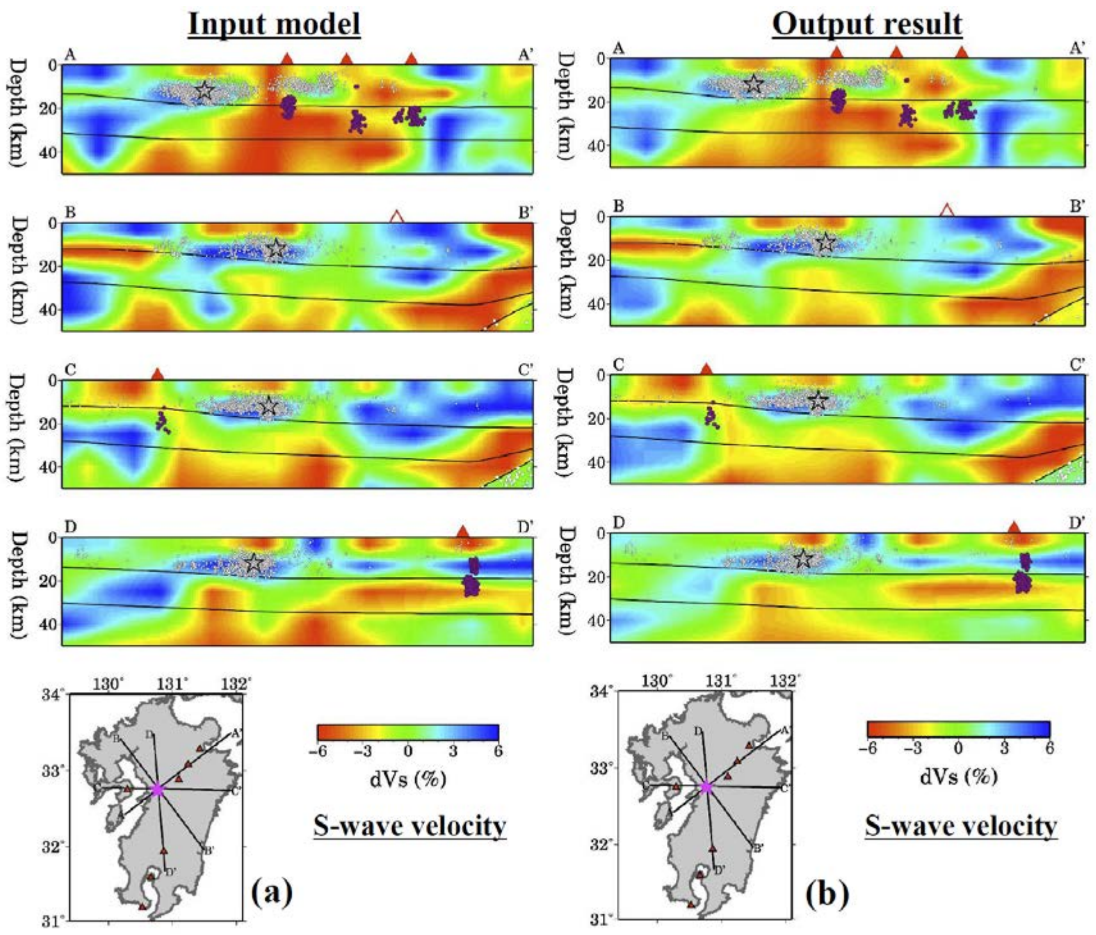

Fig. S13. The input model (a) and output result (b) of a restoring resolution test for S-wave tomography. The labels are the same as those in Fig. 5. This figure was generated using the Generic Mapping Tools version 4.5.8 (http://gmt.soest.hawaii.edu).

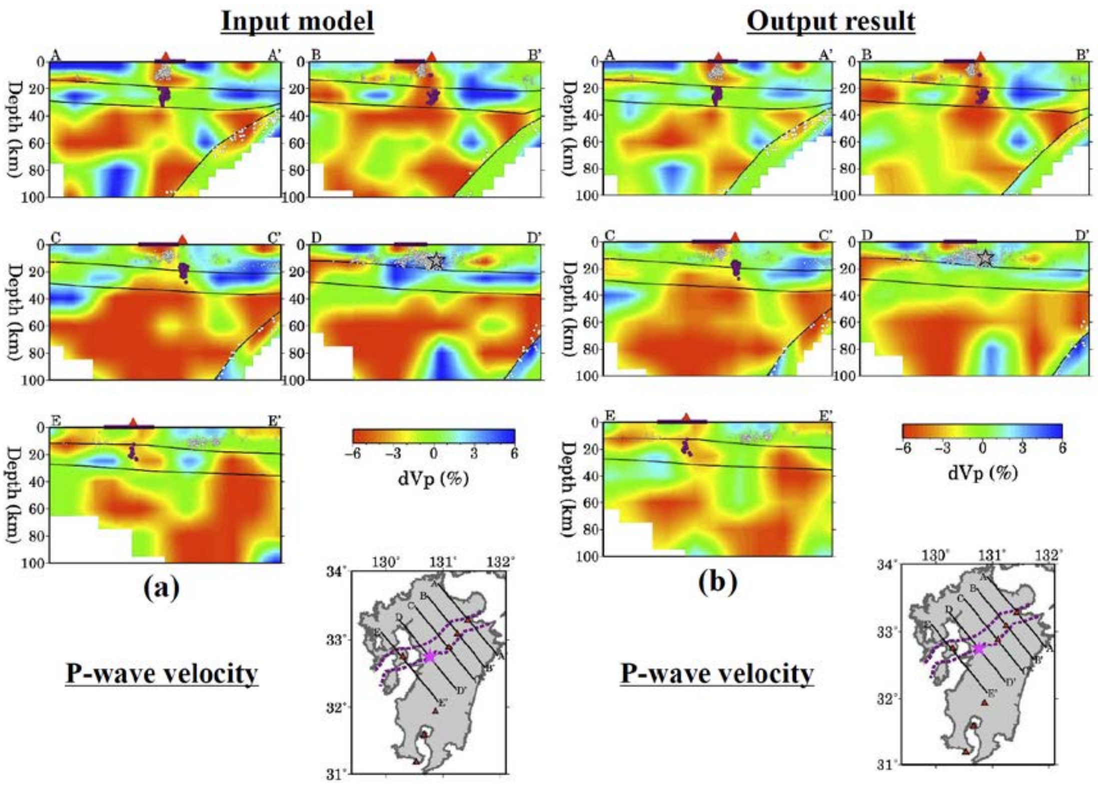

Fig. S14. The input model (a) and output result (b) of a restoring resolution test for P-wave tomography. The labels are the same as those in Fig. 6. This figure was generated using the Generic Mapping Tools version 4.5.8 (http://gmt.soest.hawaii.edu).

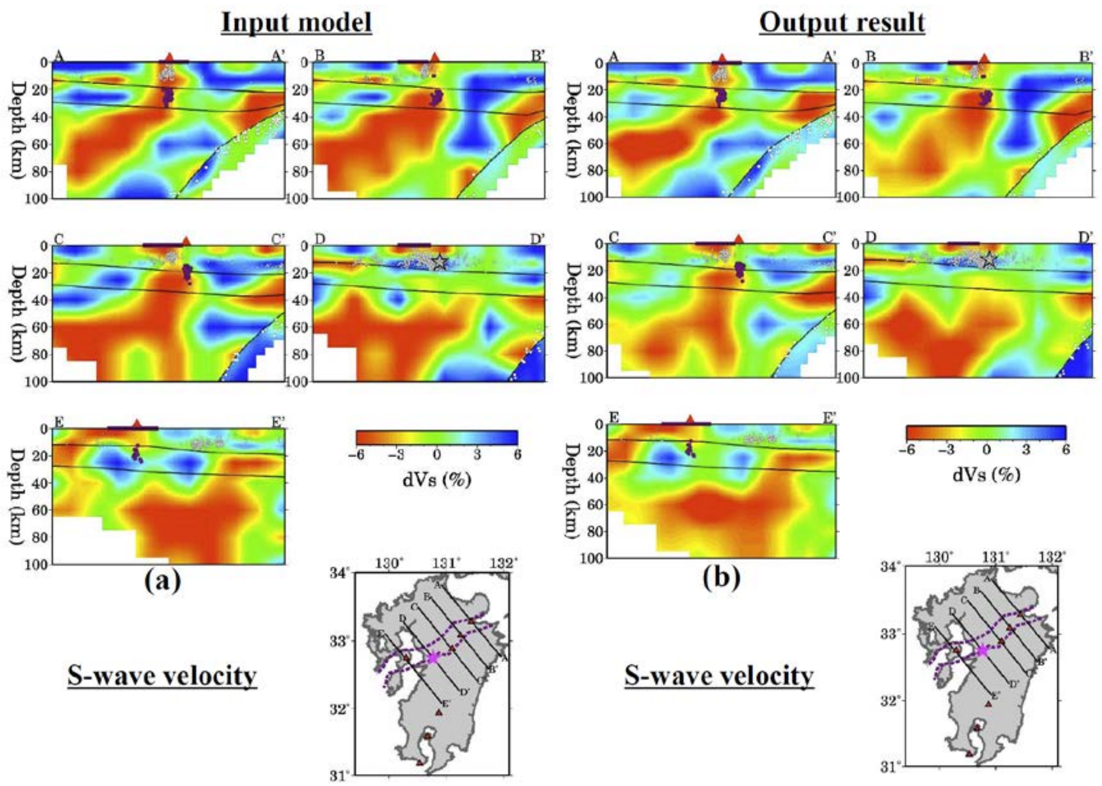

Fig. S15. The input model (a) and output result (b) of a restoring resolution test for S-wave tomography. The labels are the same as those in Fig. 6. This figure was generated using the Generic Mapping Tools version 4.5.8 (http://gmt.soest.hawaii.edu).
